# 3D printed CT-based abdominal structure mannequin for enabling research

**DOI:** 10.1186/s41205-020-0056-9

**Published:** 2020-02-05

**Authors:** Vahid Anwari, Ashley Lai, Ali Ursani, Karina Rego, Behruz Karasfi, Shailaja Sajja, Narinder Paul

**Affiliations:** 1 Joint Department of Medical Imaging, University Health Network, Toronto, Ontario Canada; 2 University of Toronto, Toronto, Ontario Canada; 3 Western University, London, Ontario Canada; 4 Quantitative Imaging for Personalized Cancer Medicine (QIPCM) Advanced Imaging Core Lab, Techna Institute, University Health Network, Toronto, Ontario Canada; 5 Department of Medical Imaging, London Health Sciences Centre, London, Ontario Canada

**Keywords:** 3D printing, Anthropomorphic, Phantom, Modular, Medical education, Tissue mimicking, Computed tomography, CT, Molding, Medical imaging

## Abstract

An anthropomorphic phantom is a radiologically accurate, tissue realistic model of the human body that can be used for research into innovative imaging and interventional techniques, education simulation and calibration of medical imaging equipment. Currently available CT phantoms are appropriate tools for calibration of medical imaging equipment but have major disadvantages for research and educational simulation. They are expensive, lacking the realistic appearance and characteristics of anatomical organs when visualized during X-ray based image scanning. In addition, CT phantoms are not modular hence users are not able to remove specific organs from inside the phantom for research or training purposes. 3D printing technology has evolved and can be used to print anatomically accurate abdominal organs for a modular anthropomorphic mannequin to address limitations of existing phantoms. In this study, CT images from a clinical patient were used to 3D print the following organ shells: liver, kidneys, spleen, and large and small intestines. In addition, fatty tissue was made using modelling beeswax and musculature was modeled using liquid urethane rubber to match the radiological density of real tissue in CT Hounsfield Units at 120kVp. Similarly, all 3D printed organ shells were filled with an agar-based solution to mimic the radiological density of real tissue in CT Hounsfield Units at 120kVp. The mannequin has scope for applications in various aspects of medical imaging and education, allowing us to address key areas of clinical importance without the need for scanning patients.

## Introduction

Since the discovery of x-rays in 1895, major advances have taken place in X-ray imaging including computed tomography (CT), dual energy (DE) imaging, cone beam CT (CBCT) and digital tomosynthesis (DT) [1–6]. Because these radiologic imaging technologies have been shown to expose the patient to harmful ionizing radiation, rigorous quality assurance (QA) testing is needed to minimize the radiation dose and maximize the diagnostic information from each scan [7]. This process requires careful tailoring of the exposure parameters to the diagnostic task required and to the patient body habitus [8, 9]. An anthropomorphic X-ray phantom is a radiologically accurate and realistic model of the human body. Anthropomorphic phantoms have been used to provide realistic QA testing of medical imaging technologies and can be used to test new imaging protocols for radiation exposure, absorbed dose and effective dose [10]. Anthropomorphic phantoms have also been used for education and training of imaging professionals in the operation of imaging equipment. However, current commercially available and research phantoms have significant limitations. Many phantoms are very expensive [11]. Some anthropomorphic phantoms designed for X-ray or CT imaging and equipment calibration have provided a complex, detailed imaging target but remain fixed in their structure [12–14]. Other anthropomorphic phantoms have demonstrated greater scope for multi-modality imaging, but lack anatomical detail and radiological accuracy [10, 13]. There has been a particular lack of modular anthropomorphic abdominal phantoms that allow the user to remove and replace the organs to replicate different pathologies, and if required, to place foreign bodies such as dosimeters or surgical devices inside the abdominal cavity. Advances in 3D printing technology have increased the range of possibilities in the creation of innovative models for medical purposes. This includes the creation of realistic, anthropomorphic mannequins with various properties such as removable internal organs that are anatomically realistic compared to existing phantoms. The properties of such 3D-printed model(s) (3DPMs) depend on the desired medical application. In general, there are three main considerations for the selection of materials used in 3D printing of anatomical models.

1. Structural Properties: define the shape, size and anatomical detail.
2. Mechanical Properties: define how the object responds to mechanical stress; these include strength, stiffness, and plasticity.
3. Radiological Properties: define how the object interacts with X-rays; these include the material linear attenuation coefficient and the density measurement in Hounsfield Units.

Structural and mechanical properties of 3DPMs have been important in medicine for the purpose of surgical planning. This is primarily the case in craniofacial, maxillofacial, and neurosurgical reconstructive surgery [15, 16]. When creating 3DPMs for surgical planning, it is important that they have structural and mechanical properties that mimic the original tissue.

In addition, 3DPMs have been shown to replicate a wider range of pathologies [17–22] and reduce ethical concerns [20, 22, 23] relative to cadaver and animal models for education, surgical planning and training purposes. 3DPMs have been used as a communication tool between the physician and the patient to accurately represent and demonstrate complex medical situations in order to improve the patient’s understanding of their condition [24, 25]. This can positively contribute to the informed consent process. Furthermore, the increasing use of structurally accurate 3DPMs in the training of medical students and surgical residents in very complex procedures has resulted in improved outcomes and increased confidence [14, 20, 26, 27]. However, these existing 3DPMs do not exhibit radiological properties of phantoms and cannot be used for medical imaging.

To address the current limitations of phantoms and existing 3DPMs, a radiologically tissue realistic and modular anthropomorphic abdominal model was designed and constructed using existing medical imaging data and low-cost 3D printing. Five abdominal organs were printed; the liver, spleen, both kidneys, the small and large bowel. The organs were of approximate dimensions for a healthy 70 kg male patient and the X-ray attenuation properties were comparable to human tissues at 120kVp. These organs were then used to build a modular, radiologically realistic anthropomorphic abdominal model.

This manuscript will describe the design and construction of a CT-based abdominal structure mannequin for enabling research (CASMER) with the use of 3D printing techniques to create accurate organ shells and the selection of additional packing material to achieve the desired radiological and anatomical properties. To the knowledge of the authors of this article, this is the first paper to discuss the techniques employed in this approach.

## Methods & Materials

Four different techniques were involved in creating CASMER: 1) tissue realistic 3D printing of abdominal organs, 2) material based molding of the pancreas, 3) beeswax sculpting of abdominal fat and 4) the use of off-the-shelf components for the bony skeleton and the outer shell. Almost all of the abdominal organs were 3D printed. The HU values of the abdominal organs were determined by placing several 10mm^2^ regions of interest in the abdominal viscera of 20 adults (10 males) with normal abdominal CT scans using an X-ray tube setting of 120kVp to determine mean (SD) HU values. The muscle and fat sections of the abdominal wall were sculpted from Clear Flex® urethane rubber (Smooth-ON, PA) and modeling beeswax respectively. We selected a variety of materials that had comparable atomic numbers to the principle attenuating tissue in the body organ of interest. All the materials underwent CT scanning using an X-ray tube setting of 120kVp. The materials that were selected closely mimicked the range of Hounsfield Unit (HU) values of the respective in vivo organs and tissues.

A) Mannequin shell

**Fig. 1:**
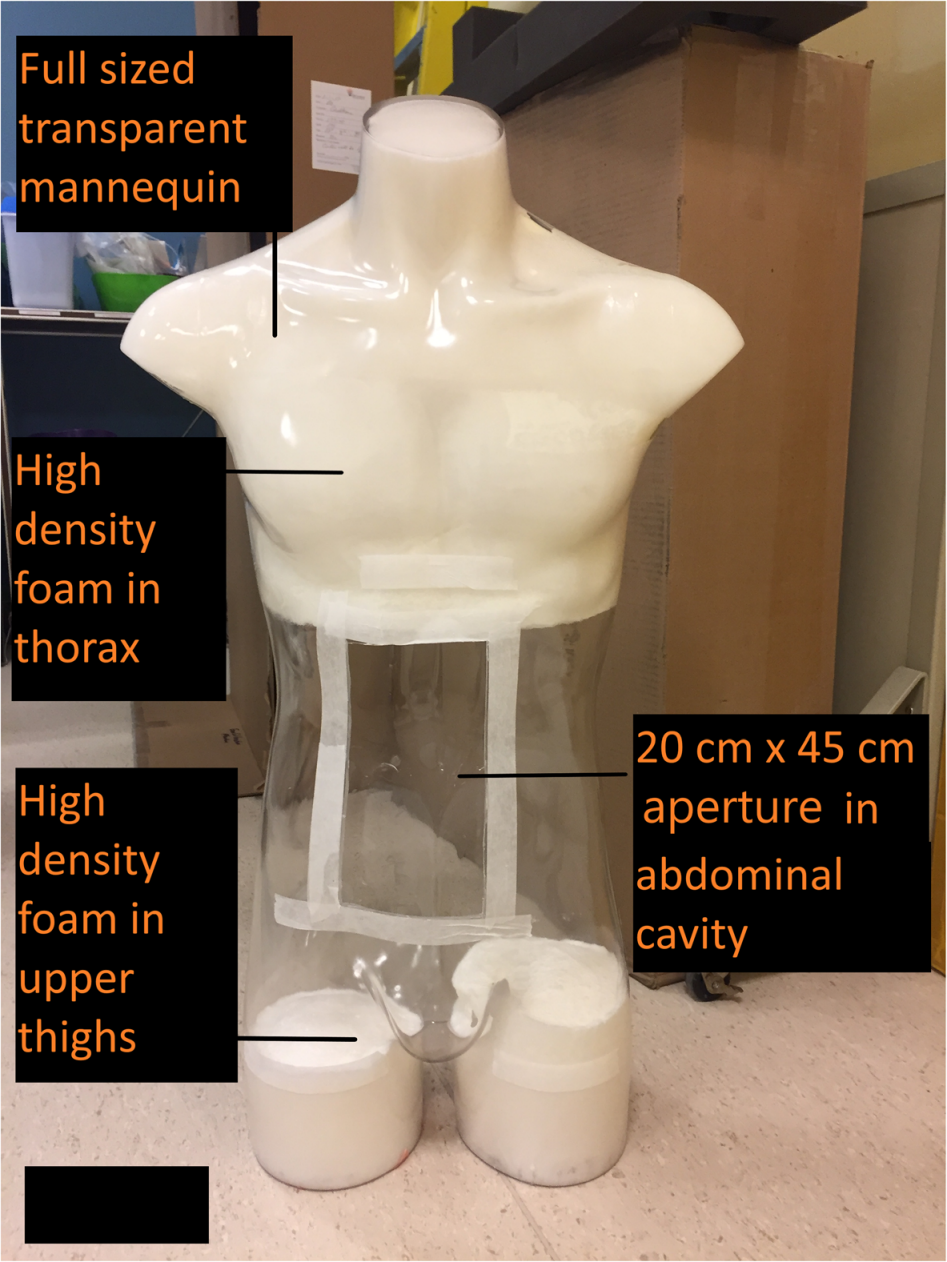
Demonstrates the transparent mannequin shell selected to house the 3D printed organs and other structures

A hollow polycarbonate full body mannequin was used to house the 3D printed organs, pancreas, bones, muscles and surrounding adipose tissue. A 20 cm by 45 cm rectangular aperture was created in the anterior “abdominal wall” of the mannequin. The thorax and upper thighs of the phantom were filled with high density liquid urethane foam (FlexFoam-IT!® 25 series, Smooth-ON, PA) as shown in Fig. 1. The polycarbonate shell was confirmed to minimally attenuate the X-ray radiation from the CT scan, and was transparent to visible light, which facilitated visualization of the internal structures during phantom manufacture and testing.

B) 3D printing of the abdominal organs

The first step in developing 3D printed abdominal organs was to design the logistics of the 3DPM generation and fabrication method. 3D printing of these anatomically accurate organ models was a multi-step process that required input from various members of the multi-disciplinary team: radiologists, technologists, physicists and biomedical engineers [21]. In this phase, the desired outcome goals were identified, including the material properties of the target organs. 3D printing material(s) were also identified based on the anatomical organs of interest. Finally, the 3D printer was selected from the wide selection of commercially available printers. The choice of printer was dependent on several factors including the type of printing material, financial constraints, the estimated volume of the printed product and the desirable complexity including the resolution of the printed product. A 3D model experiences no loss of accuracy provided that the printer resolution is higher than the resolution of the scan that was used to acquire the image data [28].

The second step of the 3D printing process was image acquisition. Raw image data in the form of DICOM images from various sources such as computed tomography (CT), magnetic resonance (MR) and ultrasound (US) were acquired and converted to 3D print files [20, 28, 29]. CT images are most commonly used due to their intrinsic high contrast, signal-to-noise ratio, and spatial resolution that improve the differentiation of structures and facilitate image post processing [21]. The current study used anonymized contrast enhanced CT scan data from an abdominal/pelvis scan for the 3D printing process. This data was collected following approval by the Institutional Research Ethics Board (REB).

**Fig. 2:**
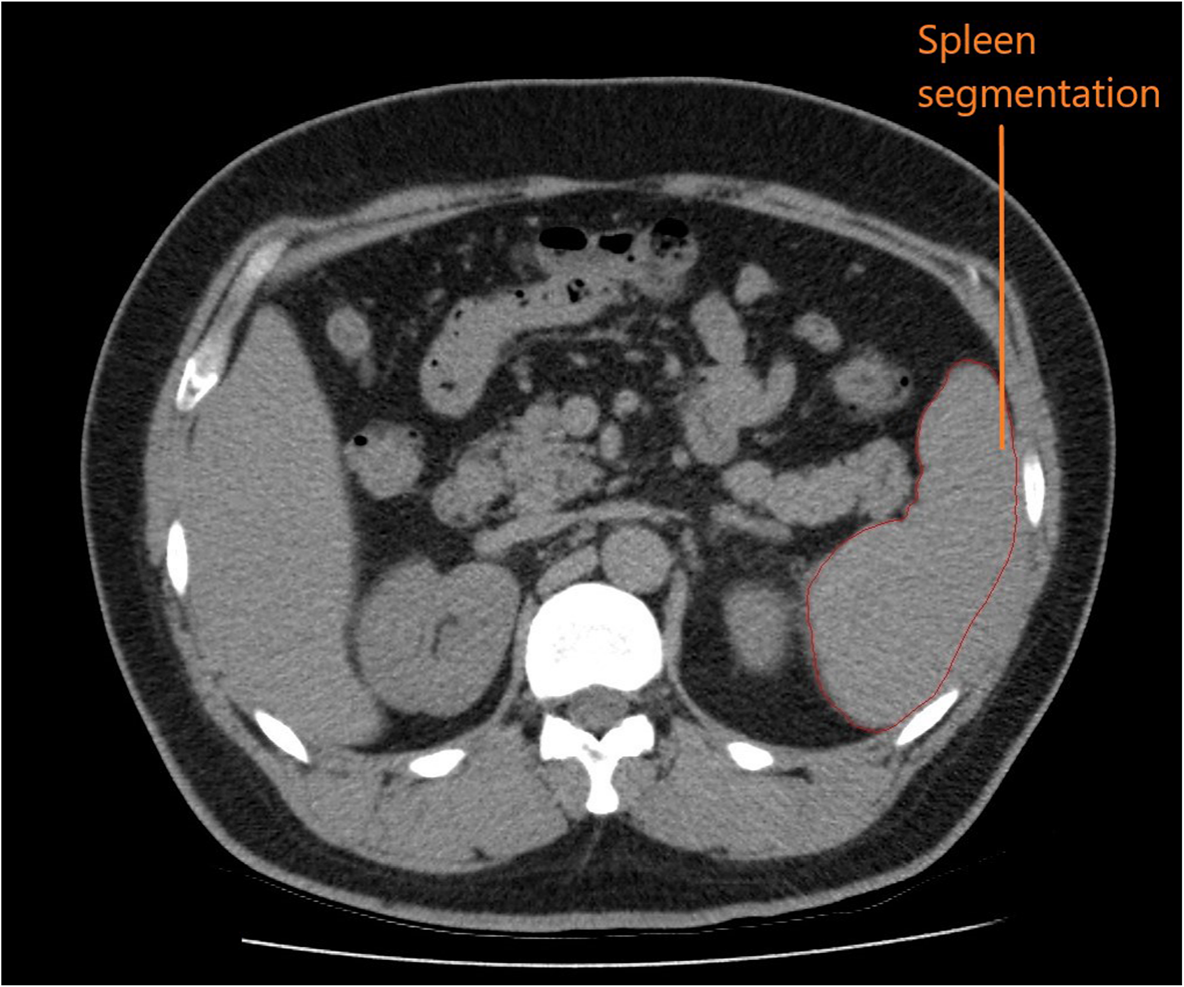
Manual segmentation was performed on the spleen and other organs using the transaxial images from the abdominal CT scan

The third step of the 3D printing process involved image segmentation of organs. A medical radiation technologist (MRT) processed the 2D source image data with segmentation software (Vitrea®, v.6.9, Vital Images, Minnetonka, MN) capable of converting the segmented data to the stereolithography (STL) file format. Several different segmentation software packages are available (commercial and open-source) for this purpose [21, 30]. Alternatively, anonymized DICOM data of a contrast enhanced scan could have been loaded into the open source Slicer software [31, 32] (v.4.7.0) and cropped to the organ of interest. In this study, both the Vitrea® software and the Slicer software (Boston, MA) were used to perform the segmentation. The complexity of the segmentation step increased as the organ complexity increased. Accurate segmentation required placing regions of interest (ROIs) around the desired tissues, either manually or automatically [33]. Each organ and its associated vasculature were manually contoured on each transaxial CT image (Fig. 2). The segmented anatomies from the DICOM data were then converted to the STL file format, which is recognized by 3D printers [29].

**Fig. 3:**
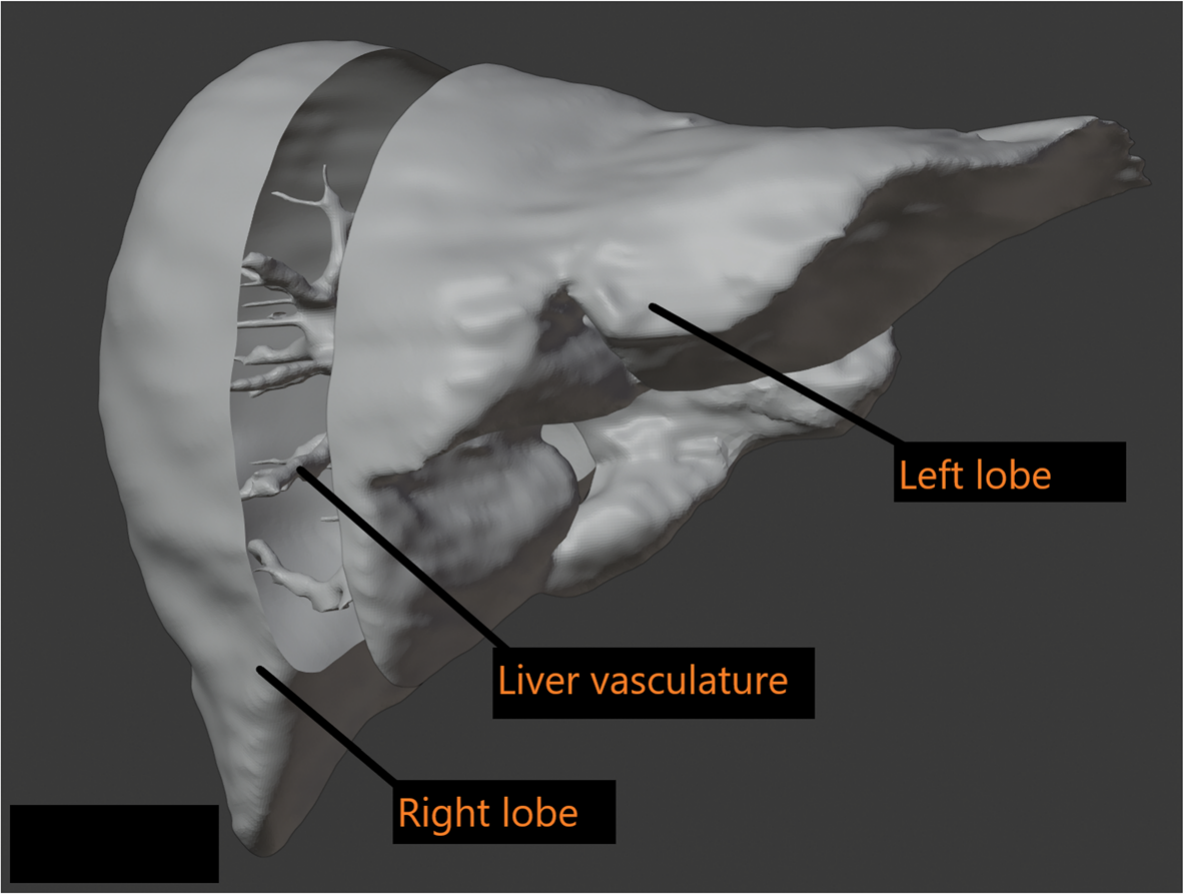
The liver was sectioned into 3 components digitally using Blender software to fit the 3D printer bed

The fourth step of the 3D printing process was image post processing. It was necessary to ensure that each model had adequate structural supports prior to printing. Hence, further editing of the file was performed to achieve an accurate and successful print. The STL file format defined surfaces as a mesh of triangles that enclose regions of space. The mesh was a series of interconnected vertices that formed the surface of the object being modeled [25]. These regions were derived from ROIs and made up the individual parts of the 3D objects to be printed. The STL files were manipulated and refined using an open source computer-aided design (CAD) software called Blender (v.2.78) (Amsterdam, NL). This process consisted of “closing” open gaps, smoothing out surfaces, and adding support structures. These alterations were necessary because 3D printers can only interpret the physical meaning of STL surfaces when ROIs are completely enclosed, and separate structures are connected to each other such that they appear to be a single structure [33]. Additional post-processing included editing of internal vessels to ensure that each vessel had a minimal wall thickness of 1 mm to provide for structural integrity during the printing process. Each organ was sectioned into smaller portions to facilitate 3D printing; the central vasculature was printed as a single piece in all organs and the outer shell was printed in 2–4 separate pieces depending on the size of the organ (Fig. 3). After the models were sectioned, the individual parts were imported into Cura (v. 15.04.5) and arranged for optimal print settings. Cura (Utrecht, NL) software ‘sliced’ the STL file into layers and generated a tool path for the print head to follow while depositing filaments. Each successive layer was built upwards, which created the 3D model.

**Fig. 4:**
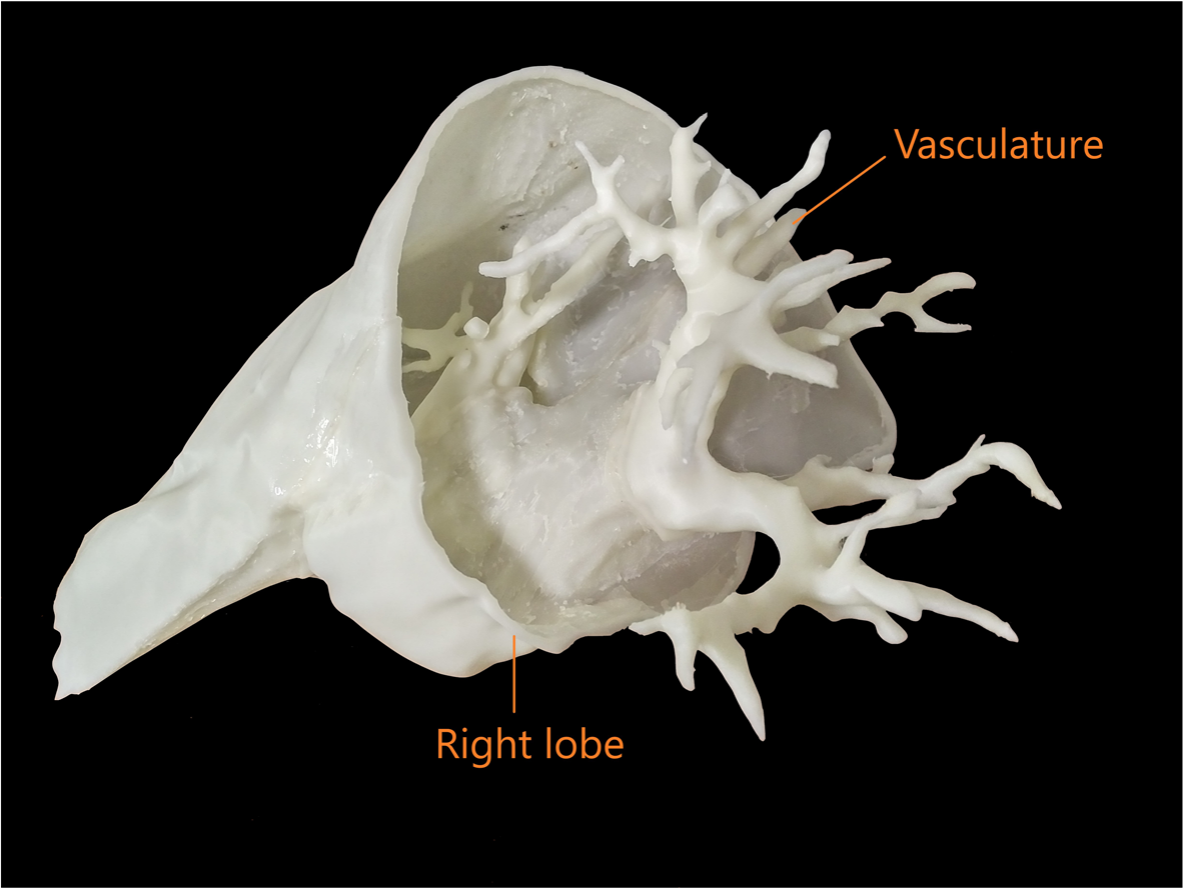
The right lobe of the liver was printed as 1 of 3 sections and joined to the vasculature

**Fig. 5:**
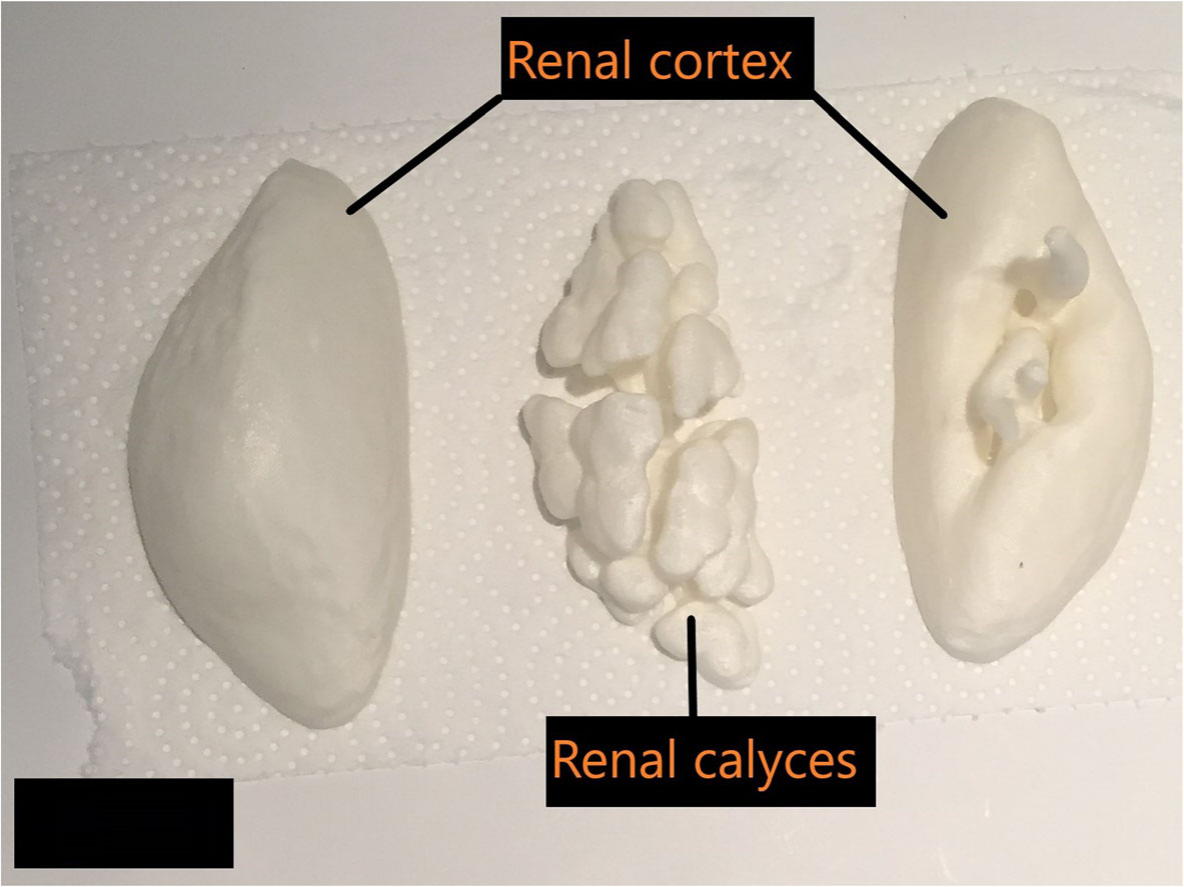
The outer renal cortex and inner calyces were separately printed as shells

The fifth step was 3D printing. A Rostock Max V2 printer using the material extrusion (fused deposition modeling) technique was used for printing [34, 35]. Material extrusion printing uses a controlled extrusion head to successively deposit layers of the printing material onto the build platform. The printer was equipped with a 0.5 mm nozzle, having a minimum layer height of 0.0125 mm and an X and Y-axis resolution of 0.1 mm. The layer height was set to 0.2 mm for increased speed of printing, providing a Z resolution of 0.2 mm (200 μm). This setting was chosen because the liver and large colon organ shells were too large and would not have fitted the printing tray as a single piece. Therefore, the organs were printed in several sections, and a 0.2 mm layer height provided a more reasonable print time for the different pieces (i.e. 20 h for a full liver at 0.2 mm versus 40 h at 0.1 mm layer-thickness). Secondly, since the intended purpose of the organ shells was not surgical, there was latitude in choosing a bigger layer height to achieve a reasonable printing time. Additionally, the chosen printer was a consumer grade entry level machine that was calibrated to print at 0.2 mm layer thickness. Acrylonitrile butadiene styrene (ABS) plastic was the selected material for the organ shells due to the material’s rigid and robust structure. For trial, open-source kidney models were printed to determine the feasibility of printing with ABS [36, 37]. When printed and imaged with CT; ABS plastic had an attenuation similar to soft tissue layers of organs within the abdominal cavity. The hollow shell of the liver, kidneys, spleen and the large and small colon were 3D printed with shell thicknesses of 1–1.75 mm using ABS filament. The example of the liver and kidney are provided in Figs. 4 and 5 respectively.

C) Clinical Use Preparation

**Fig. 6:**
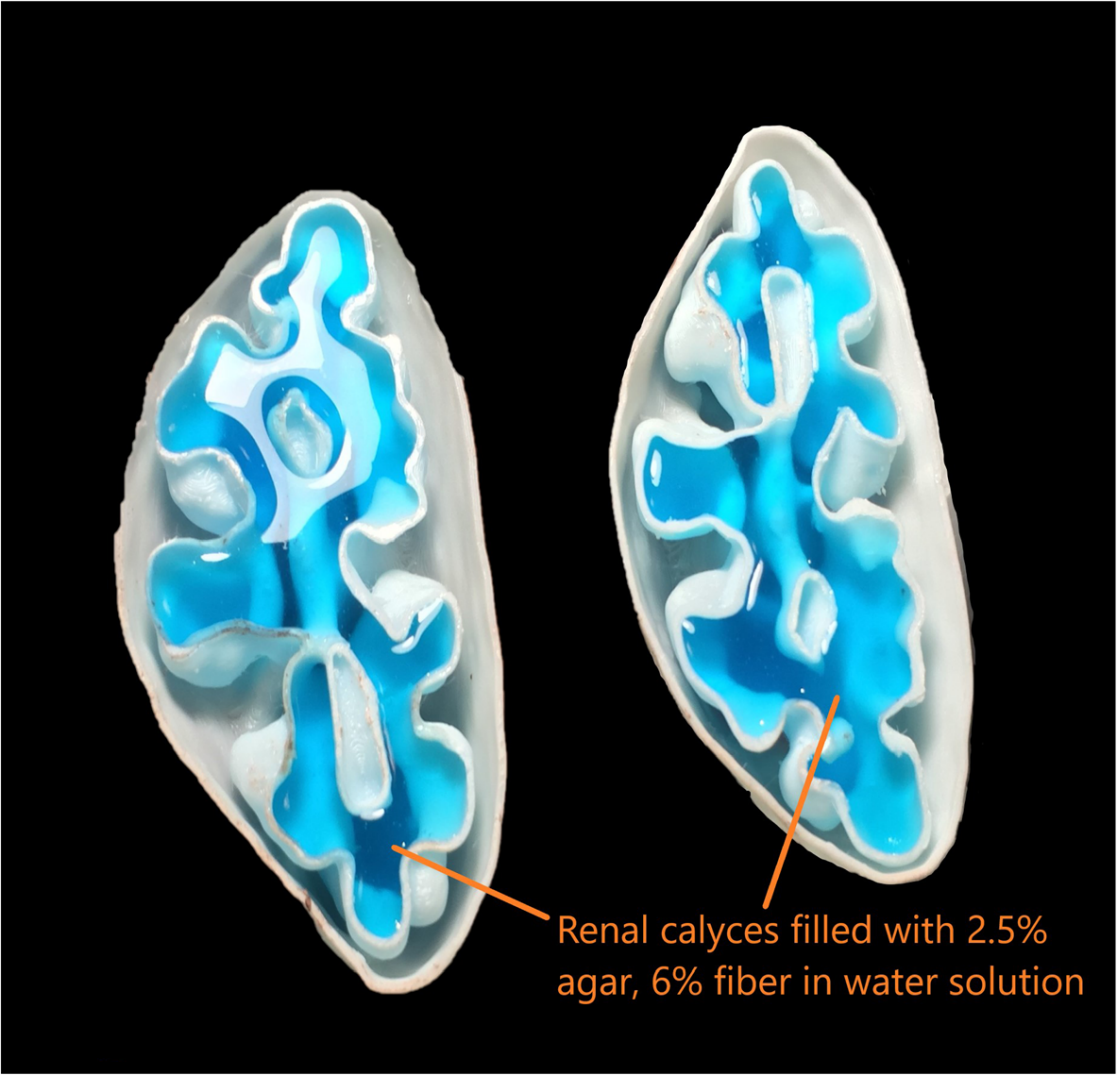
A cross-sectional view of the internal cavities of the two halves of the kidney demonstrates filling with agar solution (light blue) for a radiological match

The first step in clinical use preparation was to fill the hollow 3D printed cavities of the organs with attenuating material to simulate the biological material which fills these cavities in the human body. The 3D printed central vasculature of each abdominal organ was injected with iodinated contrast media diluted with normal saline to mimic the attenuation of the vessels in vivo at 120kVp. To achieve the attenuation of vessels in the abdomen (HU of 90.0 ± 2.5), 10.0 mL of iodine contrast was diluted into a mixture of 1.5 l of saline solution, 2.5% agar and 3.5% soluble fiber [38, 39]. After pouring, the injection site was sealed with polyurethane rubber adhesive to trap the contrast media and saline solution. Water soluble agar was chosen to fill the 3D printed organ cavities. Through experimentation, it was determined that 2.5% agar in distilled water has a mean HU of 11.4 (±5.2). We required higher HU values for abdominal organs, as set out by previously published literature [40]. However, adding increasing amounts of the agar concentration into distilled water resulted in a higher viscosity. This was undesirable as a more viscous agar solution would not have filled the organ cavities fully. To keep the solution less viscous and to raise the HU value of agar, soluble fiber was added to the mixture. Addition of 12% soluble fiber into 2.5% agar and water raised the attenuation of the mixture from 11.4 (±5.2) to 40.0 HU at 120kVp. To achieve an x-ray attenuation of 20 HU, 6% soluble fiber was poured into 2.5% agar and water. To fill the 3D printed organ shells with the agar, distilled water and fiber solution, a 250 mL syringe was inserted into a small opening in the organ shell. A cross section of the 3D printed kidney shells filled with agar solution is demonstrated in Fig. 6.

These steps were followed for all of the organs that were printed as shells including the kidneys, spleen and the colon (Table 1). The liver parenchyma required addition of 0.3% soluble iodinated contrast media to raise the CT attenuation to the required threshold.

**Table 1:** 3D Printed organs and their components

Organ	Material(s)
*Liver*	*Shell*: 1 mm ABS *Parenchyma*: 1.5 L distilled water, 2.5% agar, 3.5% soluble fiber, 0.3% soluble iodinated contrast media
*Liver Vasculature*	*Shell*: 1 mm ABS *Blood mimicking solution*: 1.5 L saline, 2.5% agar, 3.5% soluble fiber 0.3% aqueous iodine
*Kidneys*	*Shell*: 1 mm ABS *Outer Cortex***:** 300 mL distilled water, 2.5% agar, 12% soluble fiber *Inner Calyces***:** 350 mL distilled water, 2.5% agar, 6% soluble fiber
*Spleen*	*Shell*: 1 mm ABS *Parenchyma*: 500 mL distilled water, 2.5% agar, 6% fiber
*Colon*	*Shell*: 1 mm ABS *Inner features*: air bubbles, 2.5% agar and corn cob filler
*Pancreas*	Flexible Urethane Rubber (Smooth-ON, Clear Flex™ 50 Series)
*Intra-abdominal Fat*	*Modeling Beeswax*: mixture of beeswax, olive oil and lanolin cream
*Muscle layers*	Flexible Urethane Rubber (Smooth-ON, Clear Flex™ 50 Series)

The second step in clinical use preparation was to add additional abdominal structures to the mannequin including bones, muscle layers and fat sculpting. To minimize 3D printing costs, we purchased pre-manufactured, radiopaque bony structures, molded the major abdominal muscles using flexible urethane liquid rubber and used modelling beeswax to mimic the intra-abdominal fat.

The bony pelvis was purchased (ORTHObones, 3B Scientific, Georgia, US) and a complete synthetic lumbar spine was also purchased (Sawbones Company, Vashon Island, WA). Both were confirmed to be of CT attenuation similar to the human skeleton at 120kVp before being placed within the mannequin. The psoas muscles were mimicked with a clear, flexible urethane liquid rubber called ClearFlexTM 50 (Smooth-ON, Macungie, PA). This liquid rubber required mixing two component parts at room temperature onto the desired surface; curing time was approximately 24 h. The same material was used to mimic the multifidus and the erector spinae muscles.

**Fig. 7:**
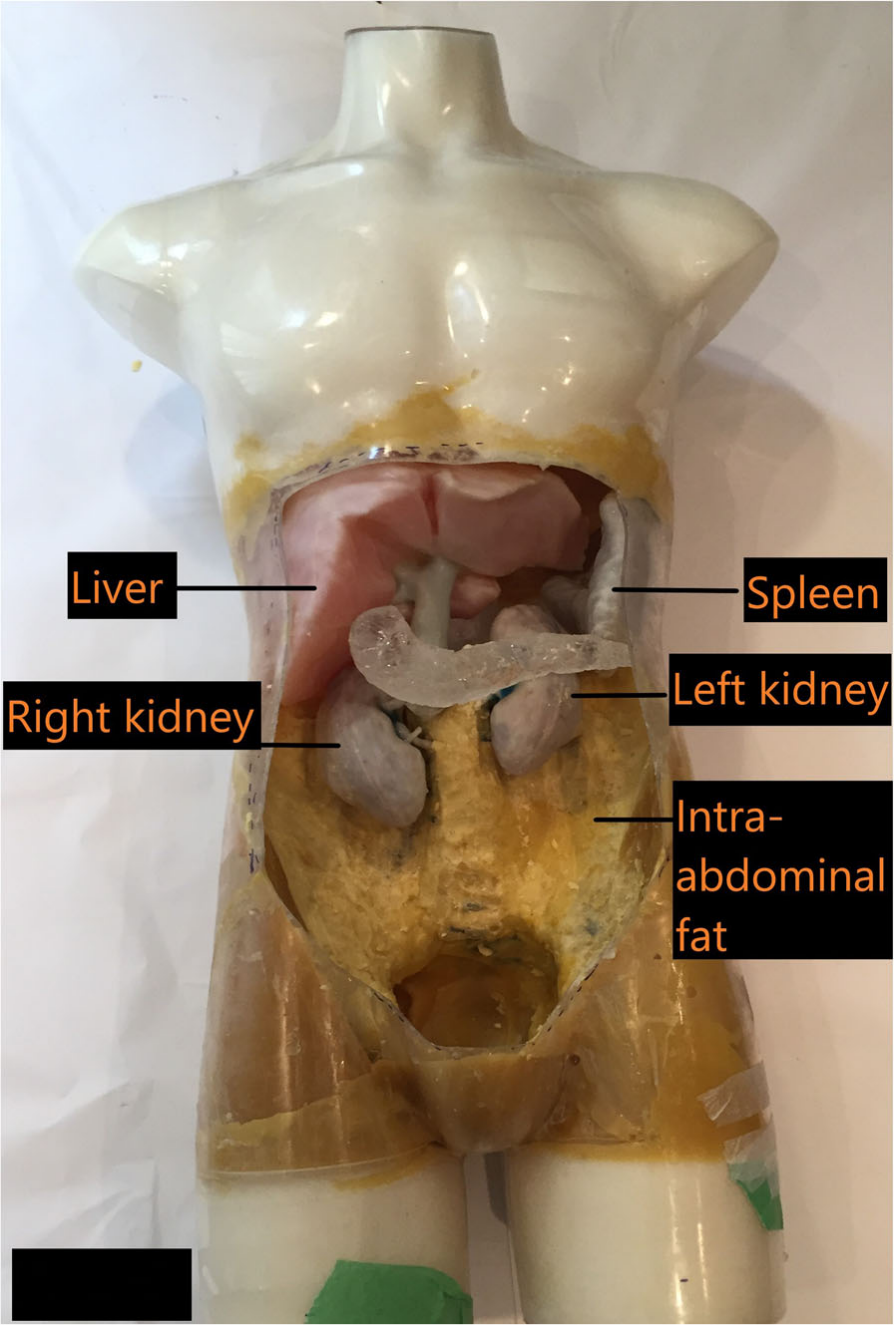
Intra-abdominal fat was mimicked with beeswax and formed a secure agent to house the removable 3D printed organs

The final component of the phantom involved using a radiologically accurate and flexible material to mimic fatty tissue. By experimenting with several materials, including vegetable oils, candle wax and plastic, it was determined that modelling beeswax was the ideal solution to mimic fat. Beeswax is a natural wax; its properties include hydrophobicity and malleability at room temperature. It has a low melting point in the range of 60–64 °C, which made it easier to melt on a stovetop. Modeling beeswax with a mixture of olive oil and lanolin cream created a soft dough textured modelling substance that liquefied when heated lightly and solidified at room temperature. This allowed the use of modeling beeswax to mimic intra-abdominal fat and provide structural support to stabilize the removable intra-abdominal organs within the mannequin as shown in Fig. 7.

## Results

**Fig. 8:**
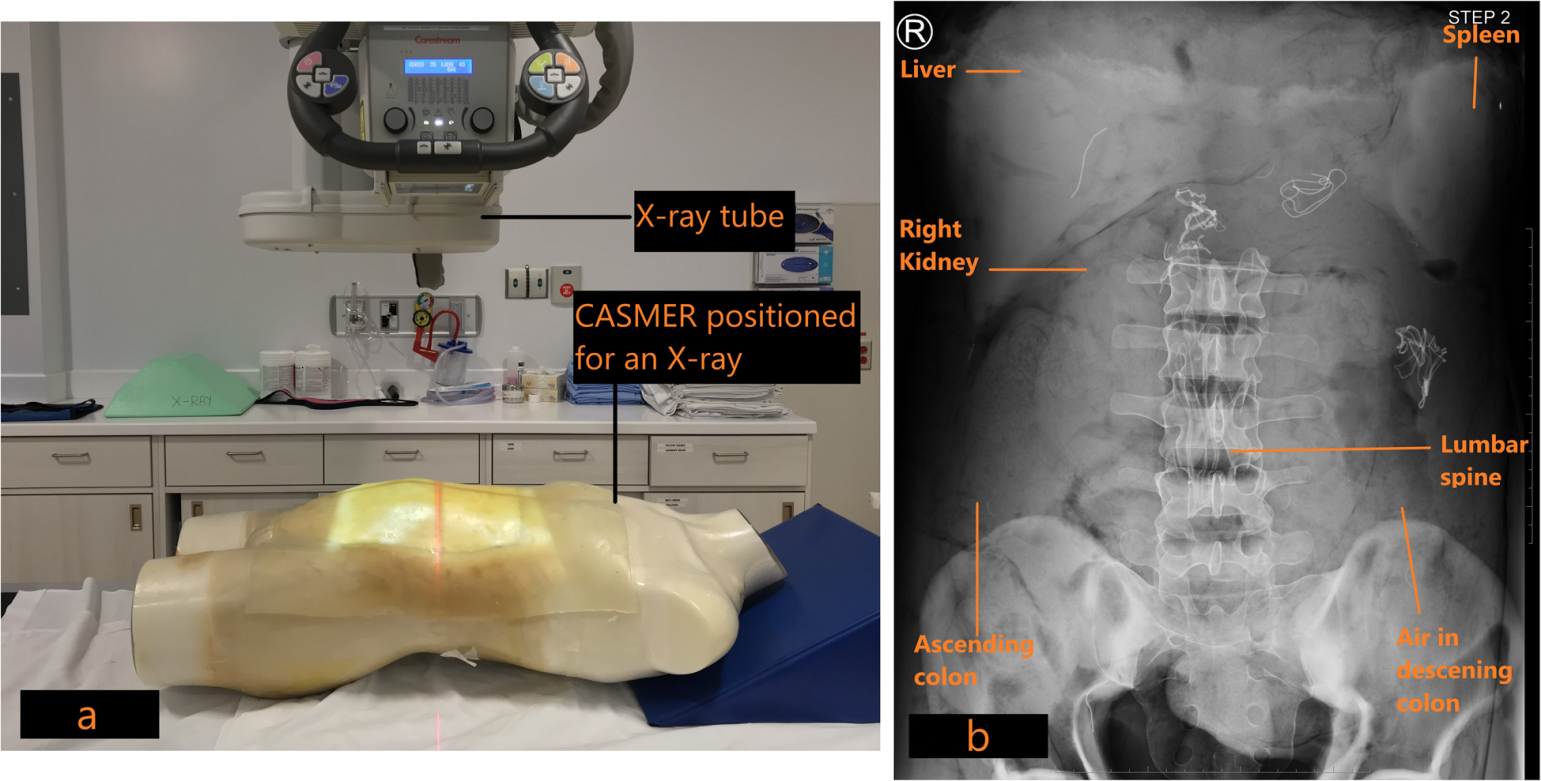
**a**: CASMER was positioned for an anteroposterior abdominal radiograph to determine radiological density. **b**: Anteroposterior X-ray of CASMER demonstrates the 3D printed organs and other structures as labelled

**Fig. 9:**
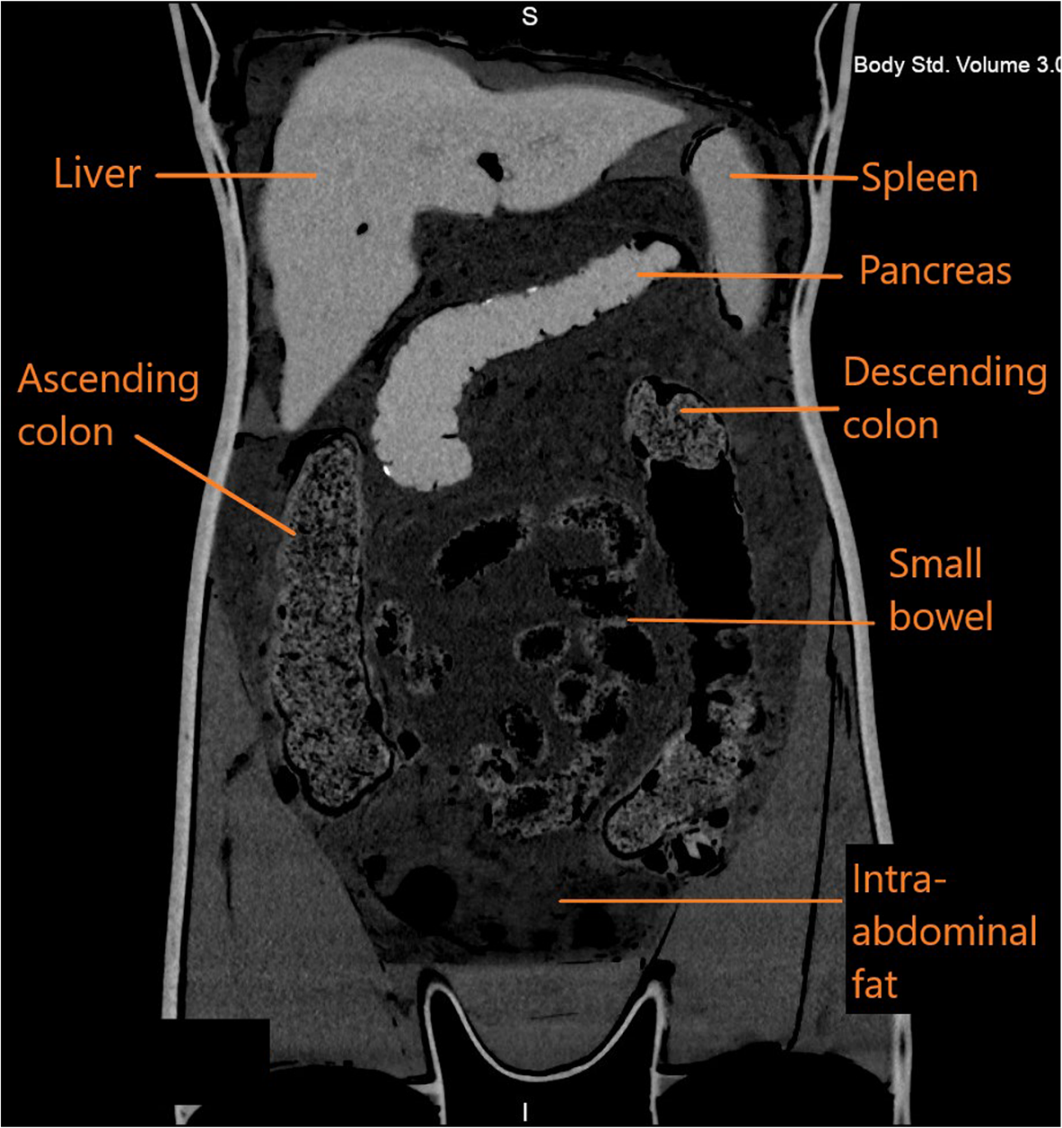
CT coronal view of CASMER demonstrates the positioned organs and surrounding intra-abdominal fat as labelled

**Fig. 10:**
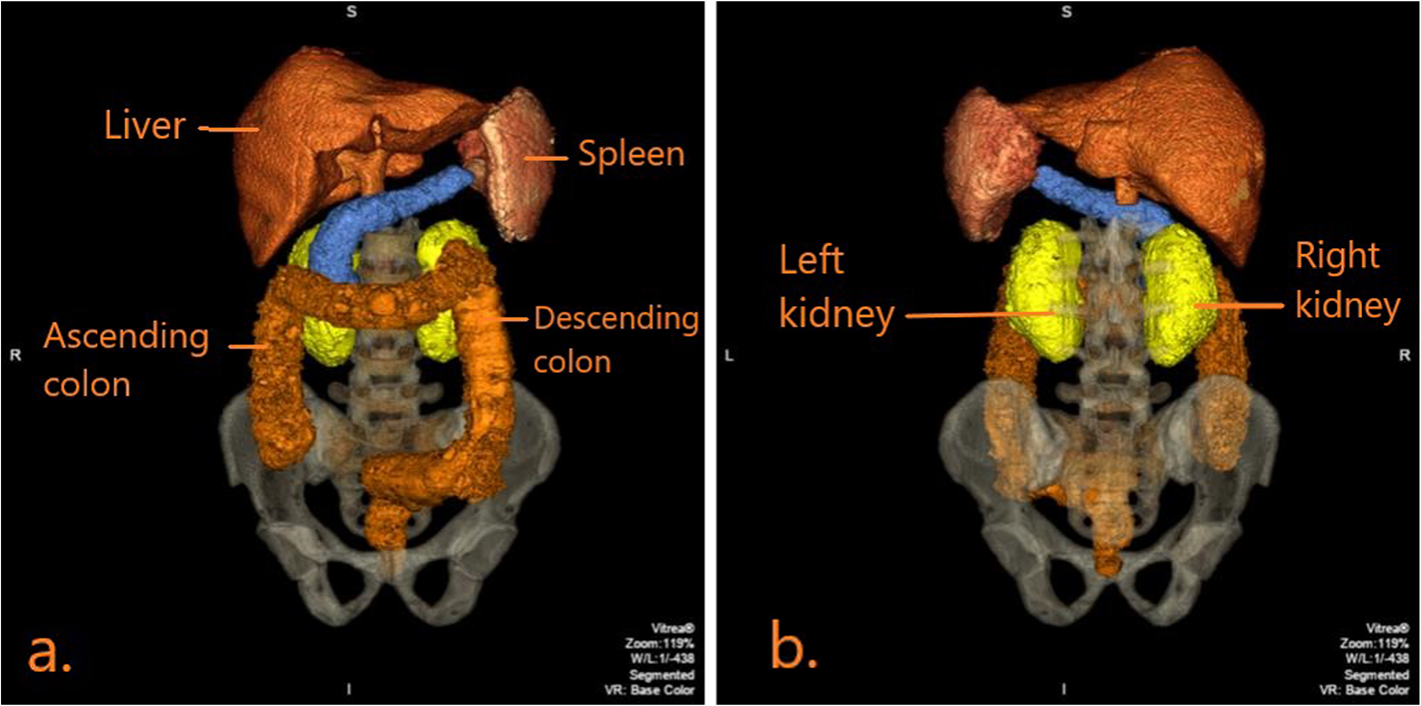
**a**: Anteroposterior view of the volume rendered image of CASMER shows the labelled 3D printed organs with correct anatomical positioning. **b**: Posteroanterior view of the volume rendered image of CASMER shows the left and right kidneys

When the construction of the mannequin was complete, CT and X-ray scans were acquired to determine the radiological accuracy of the materials inside (Table 2). Figure 8a demonstrates the positioning of the mannequin for an anteroposterior (AP) radiographic view. The resulting radiographic image is shown in Fig. 8b. Figure 9 demonstrates a coronal view of the mannequin acquired with a CT scanner (Canon Medical Systems, Otawara, JP) using an abdominal clinical protocol at 120 kVP. Figure 10a, b demonstrates volume rendered images of the 3D printed organs (except the pancreas) using the Vitrea® software.

**Table 2:** Measured Hounsfield Units of phantom components at 120kVp

Organ	Hounsfield Unit (Mean, SD)
Liver	55.0 ± 18.0
Liver Vasculature	70.0 ± 15.0
Kidneys	40.0 ± 11.0 (inner), 55.0 ± 11 .0(outer)
Spleen	40.0 ± 15.0
Colon	Too variable to measure
Pancreas	60.0 ± 14.0
Intra-abdominal fat	− 100 ± 15.0
Muscle layers	50 ± 10.0

## Discussion

This manuscript outlined the specific steps involved in the manufacturing of a 3D printed, anthropomorphic, abdominal model using CT-based scans with radiologically accurate tissue characteristics. Table 3 lists the cost of materials, scanning and labour in the development of the model. Depending on the desired characteristics and the intended purpose of a model, certain steps in the 3D model’s preparation are more important than others. For example, educational models require structural accuracy. If the sole purpose of the 3D model is to educate patients about their disease, image post processing (i.e. segmentation) is the most important step to ensure that the anatomy of the model closely resembles the actual organ. Surgical models require accuracy in physical properties in addition to structural accuracy.

**Table 3:** Manufacturing costs

Item	Amount (CAD)
Raw materials: agarose gel, fiber, beeswax, synthetic bones, ABS plastic filament	$2200
Technologist time for segmentation, organ file processing, post print clinical use preparation	$1600
3D printing of organs (liver, colon, kidneys, spleen)	$900
CT scanning time	$300
3D Printer (Rostock Max V2)	$1400
Total	$6400

For the 3D printed organ shells in this study, structural accuracy was not as important as radiological accuracy. To fill the organ shells, several materials were studied in terms of radiodensity; the chosen materials closely mimicked human tissue with respect to radiological properties. Careful review by a radiologist throughout the process ensured that the final model matched the required radiological representation [20, 21, 28].

The quality of image acquisition was critical to performing a smooth image segmentation process [41–43]. For example, in the liver, the internal vasculature must be preserved and displayed in as much detail as possible. Many factors affect this process such as the uniform contrast opacification of liver vasculature during image acquisition, the rate and volume of contrast being administered, the exposure parameters and image reconstruction parameters [41, 42, 44, 45].

When selecting an optimal CT scan for organ segmentation, the attenuation in Hounsfield Units of the selected organs should be determined from the CT files. Typically, a standard deviation (SD) of 7–10 HU is acceptable image noise for 3D modeling and segmentation prior to 3D printing [46, 47]. This threshold of image noise applies to most conventional abdomen CT images [47]. During 3D modelling, especially for vascular edge enhancement, less image noise is desired, and to achieve this, a higher tube current is needed [42, 45]. Use of intravenous iodinated contrast media aids 3D segmentation for optimal opacification of target organs and vasculature [42]. Another parameter to consider when selecting an ideal scan for segmentation is the reconstruction algorithm (kernel) that is selected during the CT scan [28]. Low spatial frequency (“soft tissue”) reconstruction algorithms are preferred for better 3D segmentation compared to high spatial frequency (“bone”) algorithms [48, 49]. Lastly, the slice thickness should be as small as possible for ideal 3D rendering; 0.5 mm reconstructions provide a balance between acceptable image noise and adequate spatial resolution for proper rendering [50].

**Fig. 11:**
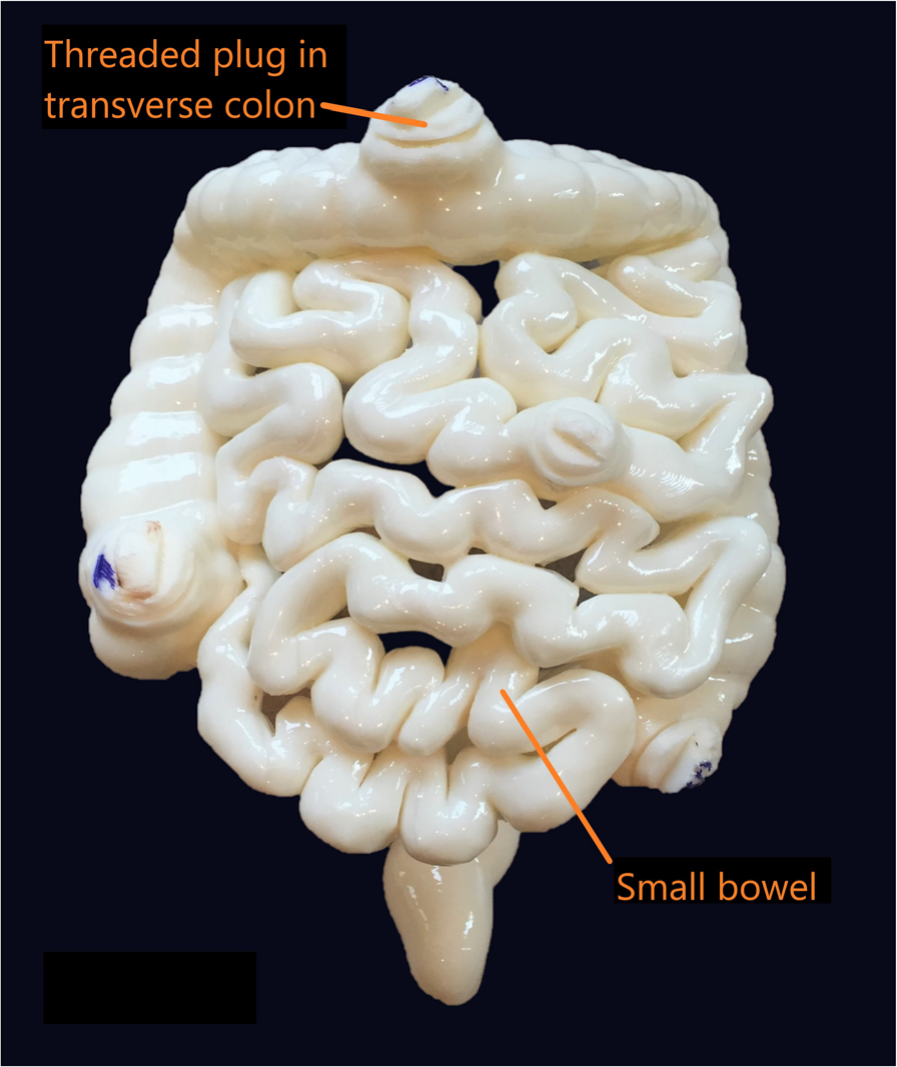
The 3D printed large colon and small bowel segments is demonstrated, with threaded plugs for internal access

In this study, the most challenging organs to segment were the small and large bowel. The CT scan data that was available was suboptimal for segmentation and 3D printing of the bowel. Therefore, the decision was made to utilize an artistic rendering of the large and small bowel that could be more easily scaled to fit within the phantom cavity. Considerable editing of the shell was necessary to make a continuous hollow channel from the gastric sphincter all the way to the anus. Four threaded plugs were also created to allow access to the interior of the bowel for the purposes of adding radiopaque material to simulate obstructions and other material normally found in the digestive tract (Fig. 11). During the post-processing component, the segmented file was converted to the STL file format.

Once a digital model was ready to be printed, a number of 3D printing parameters had to be assessed in order to determine which printing technology would be the most effective. A few important factors that were considered include printing time, availability of printers, cost of materials, color capabilities, moisture resistance, molding properties, and multi-material capabilities. After printing, the support structures that were computationally added during STL slicing were removed.

Following 3D printing, it was necessary to select an appropriate media to suspend the various organs of the abdomen. The ideal medium needed to radiologically simulate intra-abdominal and retroperitoneal fat and had to be malleable enough for removal and readjustment during placement of the 3D printed organs. After investigation with several different materials, we selected modelling beeswax to simulate intra-abdominal fatty tissue. To the knowledge of the authors, modelling beeswax has not previously been described in the manufacture of an abdominal phantom. The CT attenuation of modeling beeswax was found to be approximately − 100 HU, which is similar to abdominal fat [51].

Previous researchers have used pure safflower oil within a polyurethane mesh, commonly found as a form of air filtration material in window air conditioners [52]. The challenge with safflower oil within a polyurethane mesh is that the oil tends to sink into the bottom of the polyurethane mesh within a few hours and the polyurethane foam is not strong enough to hold the organs in place. Modeling beeswax was found to be an optimal solution for this purpose.

## Conclusion and future work

In this manuscript, the process of designing and validating a tissue realistic anthropomorphic abdominal mannequin was presented. There are several avenues for future uses of the model, some of which are mentioned below. CASMER will be available for training medical radiation technology (MRT) students in cross sectional anatomy of the abdomen and for radiation dosimetry calculations. We will also explore 3D printing of pathologies within organs to facilitate training in performing image guided procedures.

## Data Availability

Not applicable.

## Abbreviations

3DPMs: 3D printed models
ABS: Acrylonitrile butadiene styrene
CT: Computed Tomography
STL: Stereolithography
